# A diagnostic test to examine early improvement as a predictor of later response to lurasidone in bipolar depression

**DOI:** 10.1002/npr2.12319

**Published:** 2023-01-12

**Authors:** Taro Kishi, Hiroshi Nakamura, Tadafumi Kato, Nakao Iwata

**Affiliations:** ^1^ Department of Psychiatry Fujita Health University School of Medicine Toyoake Japan; ^2^ Medical Affairs Sumitomo Pharma Co., Ltd. Tokyo Japan; ^3^ Department of Psychiatry and Behavioral Science Juntendo University Graduate School of Medicine Tokyo Japan

**Keywords:** bipolar depression, diagnostic test, early improvement, lurasidone

## Abstract

**Introduction:**

Kato et al. reported results of a 6‐week, double‐blind, randomized, placebo‐controlled trial of lurasidone in adults with bipolar depression (BDep).

**Aim:**

We performed a post hoc analysis using data from the lurasidone trial to predict later responses from early improvements.

**Methods:**

An early improvement was defined as a ≥20% reduction in Montgomery–Åsberg Depression Rating Scale (MADRS) total score at Week 2; response was defined as a ≥50% reduction in MADRS total score at Week 6; symptomatic remission were defined as a score of ≤8 on MADRS total score at Week 6.

**Results:**

Both sensitivity and negative predictive value (NPV) were higher for the remission outcome than for the response outcome. The interpretation of sensitivity and NPV in the lurasidone group when remission is an outcome is as follows. It means (1) that, from all remitters at Week 6, 80.6% was identified as such at Week 2 on the basis of their early improvement and (2) that a patient showing non‐improvement at 2 weeks had 93.5% probability of being a non‐remitters at Week 6. However, the values of specificity for both response and remission in the lurasidone group were not high.

**Conclusion:**

Patients who did not show an early response at Week 2 cannot be predicted with a high probability to also show poor improvement at Week 6. In fact, some patients who did not show early response at 2 weeks might have marked improvement at 6 weeks.

## Abstract 2

We performed a post‐hoc analysis using data from the lurasidone trial to predict later responses from early improvements. Both sensitivity and negative predictive value values were higher for the remission outcome than for the response outcome.

## Graphical abstract

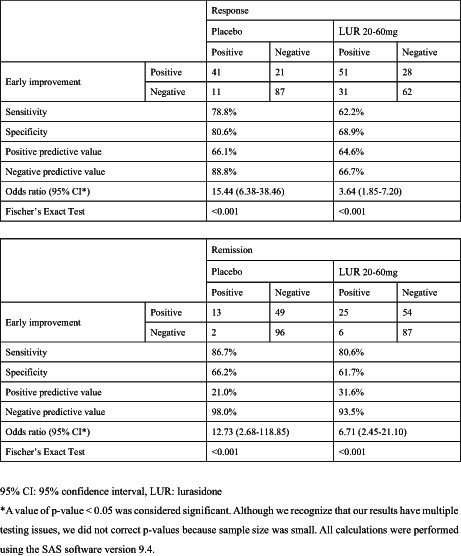

In 2020, Kato et al.^1^ reported results of a 6‐week, double‐blind, randomized, placebo‐controlled trial of lurasidone in adults with bipolar depression (BDep). The findings demonstrated that lurasidone 20–60 mg/day (LUR20–60) improved symptoms of depression and anxiety and was generally well‐tolerated. Individuals with bipolar disorder (BD) have about a 12 times higher ratio of suicide deaths to suicide attempts than the general population.^2^, ^3^ This means that individuals with BD usually employ more lethal suicide methods compared with the general population.^2^ Suicidal ideation is also about five times more frequent in individuals with BD than in the general population.^2^, ^4^, ^5^ Thus, early intervention and treatment with medications, along with close observation and follow‐up are needed to prevent suicide in individuals with BD. However, the length of time clinicians should wait before judging that a medication is not effective for BD is an unresolved clinical question. Here, we performed a post‐hoc analysis using data from the LUR trial^1^ to predict later responses from early improvements.

The detailed characteristics of patients included in the LUR trial are presented in Table S1. All data were analyzed on an intention‐to‐treat basis using the last observation carried forward method. An early improvement was defined as a ≥20% reduction in Montgomery–Åsberg Depression Rating Scale (MADRS)^6^ total score at week 2; response was defined as a ≥50% reduction in MADRS total score at week 6; and symptomatic remission were defined as a score of ≤8 on MADRS total score at week 6. We then assessed the association between early improvement at week 2 and treatment response or remission at week 6 using odds ratios (ORs) with 95% confidence intervals. Fisher's Exact Test was used to calculate the *p* values. Positive predictive value (PPV), negative predictive value (NPV), sensitivity, and specificity were also calculated for the response status at week 2 to predict a subsequent response at week 6.^7^, ^8^

We included 332 adults with BDep (male = 47.3%, mean age = 42.1 years, Table S1). The percentages of responders and remitters were shown Table 1. In the LUR20–60 treatment group, an early improvement was correlated with increased ORs for later favorable treatment response and remission (Table 1). From a clinical point of view, sensitivity and NPV are more important than specificity and PPV since the diagnostic test should mainly (1) ensure that LUR is not changed unnecessarily when the patient still has a good chance of responding (confirmed by high values of sensitivity), and (2) predict nonresponse satisfactorily (confirmed by high values of NPV). These values were higher for the remission outcome than for the response outcome. The interpretation of sensitivity and NPV in the LUR20‐60 group when remission is an outcome is as follows. It means (1) that, from all remitters at week 6, 80.6%was identified as such at week 2 on the basis of their early improvement, and (2) that a patient showing non‐improvement at 2 weeks had 93.5% probability of being a non‐remitters at week 6. However, the values of specificity for both response and remission in the LUR20‐60 treatment group were not high. As a result, patients who did not show an early response at week 2 cannot be predicted with a high probability to also show poor improvement at week 6. In fact, some patients who did not show early response at 2 weeks might have marked improvement at 6 weeks. In this case, for patients who are not responding early at week 2, clinicians may allow them to continue LUR20–60 treatment for a few more weeks. Thus, predicting treatment response at 2 weeks might be too early. Some patients also might show marked improvement after 6 weeks although we could not examine this hypothesis because we did not have the results after 6 weeks for all participants included in the trial. Another diagnostic test including other antipsychotic studies for patients with bipolar depression reported similar results to our study.^9^ The placebo group also showed high OR for the prediction of response by early improvement. This could be interpreted that if clinicians tried “no treatment” in practice (e.g., to be certain about the diagnosis), clinicians could identify placebo responders early. The results could also be explained by the higher NPV. However, because PPV of placebo group was low, it does not necessarily mean that a patient who improves early on with placebo will eventually benefit from the placebo. Such placebo response has also been observed in schizophrenia studies.^7^, ^8^, ^10^ The study had several limitations. First, a predict value decrease markedly as prevalence decreases. Because the definitions of response and remission were strict, the rates of those outcomes were low. Consequently, PPV values for remission were relatively low. Considering that the results of our study were based on a randomized trial, our results might not apply to clinical practice. Second, our study had a small sample size. Third, the lurasidone study^1^ which was used for this study had the following exclusion criteria for the participants: (1) patients scored ≥4 on MADRS^6^ Item 10 (suicidal thoughts) at screening or baseline, and (2) patients responded Yes to the Columbia Suicide Severity Rating Scale^11^ Item 4 (active suicidal ideation with some intent to act, without specific plan) or Item 5 (active suicidal ideation with specific plan and intent) at screening (within 6 months prior to screening) or at baseline, or were otherwise judged to be an imminent risk of suicide or injury to self, others, or property. Therefore, we could not investigate focusing on suicidal ideation.

**TABLE 1:** Correlations between and predictive value of early improvement at week 2 and response and remission at week 6

		Response			
		Placebo		LUR 20–60 mg	
		Positive	Negative	Positive	Negative
Early improvement	Positive	41	21	51	28
	Negative	11	87	31	62
Sensitivity		78.8%		62.2%	
Specificity		80.6%		68.9%	
Positive predictive value		66.1%		64.6%	
Negative predictive value		88.8%		66.7%	
Odds ratio (95% CI)		15.44 (6.38–38.46)		3.64 (1.85–7.20)	
Fischer's Exact Test*		<0.001		<0.001	

		Remission			
		Placebo		LUR 20–60 mg	
		Positive	Negative	Positive	Negative
Early improvement	Positive	13	49	25	54
	Negative	2	96	6	87
Sensitivity		86.7%		80.6%	
Specificity		66.2%		61.7%	
Positive predictive value		21.0%		31.6%	
Negative predictive value		98.0%		93.5%	
Odds ratio (95% CI)		12.73 (2.68–118.85)		6.71 (2.45–21.10)	
Fischer's Exact Test*		<0.001		<0.001	

Abbreviations: 95% CI, 95% confidence interval; LUR, lurasidone.

* A value of *p*‐value <0.05 was considered statistically significant. Although we recognize that our results have multiple testing issues, we did not correct *p*‐values as the sample size was small. All calculations were performed using the SAS software version 9.4.

## AUTHOR CONTRIBUTIONS

Kishi was involved in the study conception and design and performed the statistical analysis. Kishi and Nakamura performed the acquisition and interpretation of the data. All the authors wrote the manuscript. Kato and Iwata supervised the review.

## FUNDING INFORMATION

None.

## CONFLICT OF INTEREST

HN is a full‐time employee of Sumitomo Pharma Co., Ltd. Other authors declare that there are no conflicts of interest in relation to this study. We declare the following interests within the past 3 years: TKishi, TKato, and NI. TKishi has received speaker's honoraria from Sumitomo, Eisai, Janssen, Otsuka, Meiji, Tanabe‐Mitsubishi, Viatris, and Takeda and research grants from Eisai, the Japanese Ministry of Health, Labour and Welfare, Grant‐in‐Aid for Scientific Research, and Japan Agency for Medical Research and Development. TKato reports grants and personal fees from Sumitomo Pharma Co., Ltd., Otsuka Pharmaceutical Co., Ltd., Meiji Seika Pharma Co., Ltd., Shionogi & Co., Ltd., Takeda Pharmaceutical Co., Ltd., and Eisai Co., Ltd., personal fees from Taisho Pharma Co., Ltd., Mochida Pharmaceutical Co., Janssen Pharmaceutical K.K., Janssen Asia Pacific, Yoshitomiyakuhin, MSD K.K., Kyowa Pharmaceutical Industry Co., Ltd., Taisho Pharmaceutical Co., Ltd., Teijin Pharma, Viatris, Mylan N.V., outside the submitted work. He is an Editor‐in‐Chief of Psychiatry and Clinical Neurosciences (Editor in Chief), and a chair of Bipolar Disorder Committee and a member of Treatment Guidelines Committee of the Japanese Society for Mood Disorders. NI has received speaker's honoraria from Sumitomo, Eisai, Eli Lilly, Janssen, Takeda, Meiji, Tanabe‐Mitsubishi, Otsuka, and Viatris and research grants from Daiichi Sankyo, Eisai, Takeda, Sumitomo, Meiji, Tanabe‐Mitsubishi, and Otsuka.

## ETHICS STATEMENT

Approval of the Research Protocol by an Institutional Reviewer Board: N/A.

Informed Consent: N/A.

Registry and the Registration No. of the Study/Trial: N/A.

Note that the data on which this post‐hoc study is based was collected in a clinical trial with clinical trial registration number NCT01986101 in clinicaltrials.gov.

Animal Studies: N/A.

## Supporting information


Table S1.

## Data Availability

Data sharing statement is as follows: Sumitomo Pharma Co., Ltd. makes individual patient, de‐identified data sets, and associated clinical documents such as study protocol, statistical analysis plan, and clinical study report available upon request via the Clinical Study Data Request site (https://www.clinicalstudydatarequest.com/Study‐Sponsors.aspx) within 12 months of posting the study results on clininicaltrials.gov. Access is provided after a research proposal is submitted and has received approval from the Independent Review Panel and after a Data Sharing Agreement is in place. Access is provided for an initial period of 12 months but an extension can be granted, when justified, for up to another 12 months.
