# Supplementary material for: A diagnostic test to examine early improvement as a predictor of later response to lurasidone in bipolar depression
Source: Neuropsychopharmacol Rep. 2023 Jan 12;43(1):137–40. doi: 10.1002/npr2.12319 (PMC10009426; doi:10.1002/npr2.12319)
Supplement: Supplementary file 1 — Table S1. [file NPR2-43-137-s001.docx]

**Supplementary Table 1. Patient’s characteristics**

|  | Early improvement | | Non-early improvement | |
| --- | --- | --- | --- | --- |
|  | Placebo (n=62) | LUR 20-60mg (n=79) | Placebo (n=98) | LUR 20-60mg (n=93） |
| Sex: Male [n (%)] | 32 (51.6) | 39 (49.4) | 41 (41.8) | 45 (48.4) |
| Age [years, mean (SD)] | 41.2 (13.69) | 42.3 (12.17) | 41.6 (12.04) | 43.1 (13.37) |
| Race [n (%)] |  |  |  |  |
| Asian | 32 (51.6) | 40 (50.6) | 36 (36.7) | 30 (32.3) |
| White | 30 (48.4) | 39 (49.4) | 62 (63.3) | 63 (67.7) |
| Country [n (%)] |  |  |  |  |
| Japan | 22 (35.5) | 30 (38.0) | 33 (33.7) | 29 (31.2) |
| Malaysia | 4 (6.5) | 5 (6.3) | 1 (1.0) | 1 (1.1) |
| Philippines | 4 (6.5) | 2 (2.5) | 1 (1.0) | 0 |
| Russia | 12 (19.4) | 18 (22.8) | 31 (31.6) | 32 (34.4) |
| Taiwan | 2 (3.2) | 3 (3.8) | 1 (1.0) | 0 |
| Ukraine | 14 (22.6) | 18 (22.8) | 28 (28.6) | 23 (24.7) |
| Lithuania | 1 (1.6) | 0 | 1 (1.0) | 4 (4.3) |
| Slovakia | 3 (4.8) | 3 (3.8) | 2 (2.0) | 4 (4.3) |
| Height [cm, mean (SD)] | 166.19 (10.263) | 166.29 (8.400) | 168.39 (9.650) | 168.95 (8.308) |
| Weight [kg, mean (SD)] | 71.77 (15.783) | 70.95 (12.868) | 71.48 (13.066) | 74.31 (13.524) |
| Body Mass Index [kg/m^2^, mean (SD)] | 25.84 (4.583) | 25.60 (3.899) | 25.18 (4.162) | 25.99 (4.124) |
| Bipolar I Disorder History [n (%)] |  |  |  |  |
| non-rapid cycling | 52 (83.9) | 68 (86.1) | 92 (93.9) | 85 (91.4) |
| rapid cycling | 10 (16.1) | 11 (13.9) | 6 (6.1) | 8 (8.6) |
| Age at Initial Onset of Bipolar I Disorder [years, mean (SD)] | 29.1 (11.43) | 30.2 (11.23) | 29.1 (10.72) | 31.0 (12.49) |
| Duration of Bipolar I Disorder from Initial Onset to Screening [years, mean (SD)] | 12.1 (9.50) | 12.2 (10.49) | 12.5 (10.21) | 12.2 (10.62) |
| Duration of Bipolar I Depression from Onset of Current Episode to Screening [weeks, mean (SD)] | 11.6 (8.04) | 12.2 (9.63) | 13.2 (10.60) | 13.3 (8.52) |
| Number of Prior Hospitalizations for Bipolar I Disorder [n (%)] |  |  |  |  |
| ・0 | 30 (48.4) | 34 (43.0) | 29 (29.6) | 34 (36.6) |
| ・1 | 10 (16.1) | 10 (12.7) | 15 (15.3) | 5 (5.4) |
| ・2 or more | 22 (35.5) | 35 (44.3) | 54 (55.1) | 54 (58.1) |
| Number of Mood Episodes for the Consequent 12 Months, by Episode Type |  |  |  |  |
| Major Depressive Episode [mean (SD)] | 1.5 (0.78) | 1.5 (0.77) | 1.4 (0.65) | 1.4 (0.67) |
| Manic Episode [mean (SD)] | 0.4 (0.58) | 0.4 (0.56) | 0.3 (0.51) | 0.4 (0.59) |
| Mixed Episode [mean (SD)] | 0.1 (0.30) | 0.1 (0.30) | 0.1 (0.24) | 0.1 (0.29) |
| Hypomanic Episode [mean (SD)] | 0.2 (0.51) | 0.1 (0.41) | 0.1 (0.30) | 0.0 (0.20) |
| Subjects with Other Psychiatric Disorders Present [n (%)] | 1 (1.6) | 3 (3.8) | 1 (1.0) | 1 (1.1) |
| Baseline MADRS total score [mean(SD)] | 30.3 (5.50) | 30.3 (6.13) | 30.9 (4.95) | 30.6 (5.17) |
| Baseline CGI-BP-S score [mean(SD)] | 4.6 (0.67) | 4.6 (0.75) | 4.6 (0.67) | 4.5 (0.65) |
| Baseline Sheehan Disability Scale Total Score [mean (SD)] | n=52 | n=68 | n=79 | n=76 |
|  | 19.3 (4.68) | 18.7 (5.54) | 20.0 (5.36) | 19.5 (5.20) |
| Baseline HAM-A total score [mean (SD)] | 17.0 (7.61) | 17.8 (8.10) | 17.0 (5.79) | 17.2 (6.23) |
| Baseline YMRS total score [mean (SD)] | 2.9 (2.59) | 3.1 (2.60) | 2.5 (2.07) | 2.8 (2.45) |
| Baseline DIEPSS total score (excluding overall severity) [mean (SD)] | 0.3 (0.70) | 0.4 (0.92) | 0.4 (1.07) | 0.3 (0.75) |
| Use of antipsychotics before the study [n (%)] | 22 (35.5) | 24 (30.4) | 35 (35.7) | 42 (45.2) |
| Use of mood stabilizers before the study [n (%)] | 26 (41.9) | 26 (32.9) | 27 (27.6) | 25 (26.9) |
| Use of antidepressants before the study [n (%)] | 13 (21.0) | 23 (29.1) | 25 (25.5) | 21 (22.6) |
| Use of anxiolytics before the study [n (%)] | 12 (19.4) | 13 (16.5) | 17 (17.3) | 16 (17.2) |
| Use of sleeping pills before the study [n (%)] | 17 (27.4) | 18 (22.8) | 25 (25.5) | 19 (20.4) |
| Use of antiparkinson drugs before the study [n (%)] | 5 (8.1) | 7 (8.9) | 6 (6.1) | 9 (9.7) |

CGI-BP-S: Clinical Global Impressions - Bipolar version - Severity of illness, DIEPSS: Drug - Induced Extrapyramidal Symptoms Scale, HAM-A: Hamilton - Anxiety Scale, LUR: Lurasidone, MADRS: Montgomery Åsberg Depression Rating Scale, SD: Standard Deviation, YMRS: Young Mania Rating Scale
